# Supplementary material for: Modeling glioblastoma heterogeneity as a dynamic network of cell states
Source: Mol Syst Biol. 2021 Sep 16;17(9):e10105. doi: 10.15252/msb.202010105 (PMC8444284; doi:10.15252/msb.202010105)
Supplement: Supplementary file 6 — Source Data for Figure 5 [file MSB-17-e10105-s004.zip › Figure5A_sourcedata/GSEA_3017/nosigdb_stateA.GseaPreranked.1622795026485/gsea_report_for_na_neg_1622795026485.html]

Report for na\_neg 1622795026485 [GSEA]

| GS  follow link to MSigDB | GS DETAILS | SIZE | ES | NES | NOM p-val | FDR q-val | FWER p-val | RANK AT MAX | LEADING EDGE || 1 | NB\_KI\_CLUSTER18 | Details ... | 28 | -0.69 | -3.08 | 0.000 | 0.000 | 0.000 | 115 | tags=54%, list=12%, signal=59% |
| 2 | LARSSON\_STATE\_6 | Details ... | 38 | -0.62 | -3.05 | 0.000 | 0.000 | 0.000 | 208 | tags=71%, list=21%, signal=87% |
| 3 | SUVA\_AC\_PMID\_31327527 | Details ... | 24 | -0.69 | -2.96 | 0.000 | 0.000 | 0.000 | 101 | tags=58%, list=10%, signal=63% |
| 4 | RICHARDS\_NEFTEL\_CELL\_2019\_AC | Details ... | 24 | -0.69 | -2.86 | 0.000 | 0.000 | 0.000 | 101 | tags=58%, list=10%, signal=63% |
| 5 | GAROFANO\_IAVARONE\_GPM\_SUBTYPE | Details ... | 144 | -0.43 | -2.85 | 0.000 | 0.000 | 0.000 | 390 | tags=70%, list=39%, signal=99% |
| 6 | OLSEN\_MESENCHYMAL | Details ... | 26 | -0.66 | -2.83 | 0.000 | 0.000 | 0.000 | 209 | tags=69%, list=21%, signal=85% |
| 7 | VANGRONINGEN\_MES | Details ... | 81 | -0.47 | -2.83 | 0.000 | 0.000 | 0.000 | 223 | tags=52%, list=23%, signal=61% |
| 8 | DOROSZKO\_U3180MG\_CLUSTER\_5 | Details ... | 18 | -0.74 | -2.82 | 0.000 | 0.000 | 0.000 | 58 | tags=50%, list=6%, signal=52% |
| 9 | RICHARDS\_NOWAKOWSKI\_SCIENCE\_2017\_ORG\_UPREG | Details ... | 225 | -0.38 | -2.76 | 0.000 | 0.000 | 0.000 | 392 | tags=64%, list=40%, signal=83% |
| 10 | OLSEN\_SCPLIKE | Details ... | 36 | -0.58 | -2.75 | 0.000 | 0.000 | 0.000 | 293 | tags=75%, list=30%, signal=103% |
| 11 | DOROSZKO\_U3180MG\_CLUSTER\_2 | Details ... | 69 | -0.47 | -2.74 | 0.000 | 0.000 | 0.000 | 368 | tags=75%, list=37%, signal=112% |
| 12 | GANDAL\_AUTISM\_UP\_PMID29439242 | Details ... | 26 | -0.63 | -2.74 | 0.000 | 0.000 | 0.000 | 62 | tags=46%, list=6%, signal=48% |
| 13 | RICHARDS\_CAHOY\_JNEUROSCI\_2008\_ASTROCYTE | Details ... | 196 | -0.38 | -2.69 | 0.000 | 0.000 | 0.000 | 226 | tags=42%, list=23%, signal=43% |
| 14 | RICHARDS\_NOWAKOWSKI\_SCIENCE\_2017\_MGE\_RG1\_UPREG | Details ... | 70 | -0.46 | -2.67 | 0.000 | 0.000 | 0.000 | 218 | tags=50%, list=22%, signal=60% |
| 15 | RICHARDS\_ZHONG\_NATURE\_2018\_ASTROCYTES\_UPREG | Details ... | 86 | -0.44 | -2.67 | 0.000 | 0.000 | 0.000 | 262 | tags=55%, list=27%, signal=68% |
| 16 | RICHARDS\_NOWAKOWSKI\_SCIENCE\_2017\_VRG\_UPREG | Details ... | 105 | -0.41 | -2.57 | 0.000 | 0.000 | 0.001 | 323 | tags=62%, list=33%, signal=82% |
| 17 | DOROSZKO\_U3054MG\_CLUSTER\_2 | Details ... | 56 | -0.47 | -2.56 | 0.000 | 0.000 | 0.001 | 104 | tags=34%, list=11%, signal=36% |
| 18 | RICHARDS\_NOWAKOWSKI\_SCIENCE\_2017\_ASTROCYTE\_UPREG | Details ... | 135 | -0.38 | -2.55 | 0.000 | 0.000 | 0.002 | 270 | tags=50%, list=27%, signal=59% |
| 19 | GANDAL\_SCHIZOPHRENIA\_UP\_PMID29439242 | Details ... | 34 | -0.54 | -2.51 | 0.000 | 0.000 | 0.002 | 254 | tags=62%, list=26%, signal=80% |
| 20 | ALLEN\_113\_ASTROL1FGFR3FOS | Details ... | 17 | -0.65 | -2.50 | 0.000 | 0.000 | 0.002 | 56 | tags=41%, list=6%, signal=43% |
| 21 | RICHARDS\_NOWAKOWSKI\_SCIENCE\_2017\_MURAL\_UPREG |  | 103 | -0.40 | -2.50 | 0.000 | 0.000 | 0.002 | 160 | tags=33%, list=16%, signal=35% |
| 22 | RICHARDS\_CAHOY\_JNEUROSCI\_2011\_ASTRO\_MATURE |  | 55 | -0.47 | -2.50 | 0.000 | 0.000 | 0.002 | 174 | tags=45%, list=18%, signal=52% |
| 23 | GANDAL\_IBD\_UP\_PMID29439242 |  | 15 | -0.69 | -2.49 | 0.002 | 0.000 | 0.002 | 166 | tags=67%, list=17%, signal=79% |
| 24 | RICHARDS\_VERHAAK\_CANCERCELL\_2010\_CLASSICAL |  | 34 | -0.51 | -2.42 | 0.000 | 0.000 | 0.005 | 386 | tags=85%, list=39%, signal=135% |
| 25 | OUTERRADIALGLIA |  | 21 | -0.59 | -2.42 | 0.000 | 0.000 | 0.006 | 111 | tags=43%, list=11%, signal=47% |
| 26 | RICHARDS\_NOWAKOWSKI\_SCIENCE\_2017\_TRG\_UPREG |  | 190 | -0.34 | -2.41 | 0.000 | 0.000 | 0.008 | 256 | tags=44%, list=26%, signal=48% |
| 27 | POLLEN\_OUTERRADIALGLIA\_PMID\_26406371 |  | 21 | -0.59 | -2.38 | 0.000 | 0.000 | 0.009 | 111 | tags=43%, list=11%, signal=47% |
| 28 | RICHARDS\_NEFTEL\_CELL\_2019\_MES1 |  | 21 | -0.55 | -2.27 | 0.000 | 0.001 | 0.022 | 209 | tags=62%, list=21%, signal=77% |
| 29 | RICHARDS\_NOWAKOWSKI\_SCIENCE\_2017\_U1\_UPREG |  | 157 | -0.33 | -2.26 | 0.000 | 0.001 | 0.023 | 207 | tags=35%, list=21%, signal=37% |
| 30 | SUVA\_MES1\_PMID\_31327527 |  | 21 | -0.55 | -2.20 | 0.000 | 0.001 | 0.041 | 209 | tags=62%, list=21%, signal=77% |
| 31 | RICHARDS\_CAHOY\_JNEUROSCI\_2015\_ASTRO\_IN\_VIVO |  | 163 | -0.32 | -2.17 | 0.000 | 0.002 | 0.047 | 294 | tags=48%, list=30%, signal=57% |
| 32 | DOROSZKO\_U3013MG\_CLUSTER\_3 |  | 22 | -0.52 | -2.17 | 0.000 | 0.002 | 0.049 | 273 | tags=73%, list=28%, signal=98% |
| 33 | NB\_KI\_CLUSTER17 |  | 31 | -0.47 | -2.16 | 0.003 | 0.002 | 0.050 | 267 | tags=61%, list=27%, signal=81% |
| 34 | NB\_KI\_CLUSTER23 |  | 34 | -0.46 | -2.16 | 0.000 | 0.002 | 0.053 | 389 | tags=76%, list=39%, signal=122% |
| 35 | TISIG\_PMID\_32649888 |  | 22 | -0.52 | -2.16 | 0.000 | 0.002 | 0.053 | 412 | tags=91%, list=42%, signal=152% |
| 36 | ALLEN\_111\_ASTROL1\_6FGFR3ETNPPL |  | 15 | -0.59 | -2.14 | 0.002 | 0.002 | 0.060 | 246 | tags=73%, list=25%, signal=96% |
| 37 | ALMSTEDT\_VGR\_DIFF |  | 140 | -0.32 | -2.09 | 0.000 | 0.003 | 0.090 | 238 | tags=40%, list=24%, signal=45% |
| 38 | IVY\_CTPAN |  | 36 | -0.44 | -2.07 | 0.002 | 0.003 | 0.104 | 167 | tags=44%, list=17%, signal=52% |
| 39 | RICHARDS\_ZHONG\_NATURE\_2018\_MICROGLIA\_UPREG |  | 33 | -0.44 | -2.07 | 0.002 | 0.003 | 0.112 | 252 | tags=55%, list=26%, signal=71% |
| 40 | LARSSON\_STATE\_2 |  | 21 | -0.51 | -2.05 | 0.003 | 0.004 | 0.125 | 306 | tags=71%, list=31%, signal=101% |
| 41 | RICHARDS\_NOWAKOWSKI\_SCIENCE\_2017\_ENDOTHELIAL\_UPREG |  | 182 | -0.29 | -2.05 | 0.000 | 0.004 | 0.131 | 209 | tags=32%, list=21%, signal=34% |
| 42 | RICHARDS\_NOWAKOWSKI\_SCIENCE\_2017\_EN\_PFC3\_UPREG |  | 52 | -0.38 | -2.04 | 0.005 | 0.004 | 0.138 | 342 | tags=65%, list=35%, signal=95% |
| 43 | ALLEN\_112\_ASTROL1FGFR3MT1G |  | 17 | -0.54 | -2.03 | 0.003 | 0.004 | 0.140 | 69 | tags=35%, list=7%, signal=37% |
| 44 | GANDAL\_ALCOHOLADDICTION\_UP\_PMID29439242 |  | 15 | -0.56 | -2.00 | 0.007 | 0.005 | 0.180 | 54 | tags=33%, list=5%, signal=35% |
| 45 | GAROFANO\_IAVARONE\_MTC\_SUBTYPE |  | 25 | -0.46 | -1.97 | 0.003 | 0.006 | 0.242 | 368 | tags=80%, list=37%, signal=124% |
| 46 | DOROSZKO\_PERIVASCULAR\_INVASION |  | 17 | -0.52 | -1.94 | 0.007 | 0.007 | 0.286 | 191 | tags=53%, list=19%, signal=64% |
| 47 | RICHARDS\_LIDDELOW\_NATURE\_2017\_A2\_REACTIVE\_ASTROCYTES |  | 16 | -0.50 | -1.88 | 0.007 | 0.013 | 0.444 | 70 | tags=38%, list=7%, signal=40% |
| 48 | GANDAL\_BIPOLAR\_UP\_PMID29439242 |  | 21 | -0.46 | -1.87 | 0.002 | 0.013 | 0.460 | 254 | tags=62%, list=26%, signal=82% |
| 49 | RICHARDS\_NOWAKOWSKI\_SCIENCE\_2017\_MICROGLIA\_UPREG |  | 93 | -0.30 | -1.85 | 0.004 | 0.015 | 0.503 | 208 | tags=33%, list=21%, signal=38% |
| 50 | RICHARDS\_NOWAKOWSKI\_SCIENCE\_2017\_IN\_CTX\_CGE2\_UPREG |  | 45 | -0.36 | -1.83 | 0.014 | 0.017 | 0.545 | 322 | tags=58%, list=33%, signal=82% |
| 51 | SINGH\_SOX2\_DOWNREGULATED\_TRANSCRIPTS\_PMID\_28122245 |  | 23 | -0.43 | -1.81 | 0.012 | 0.019 | 0.597 | 117 | tags=35%, list=12%, signal=39% |
| 52 | RICHARDS\_NOWAKOWSKI\_SCIENCE\_2017\_EN\_PFC2\_UPREG |  | 138 | -0.27 | -1.80 | 0.005 | 0.019 | 0.617 | 342 | tags=51%, list=35%, signal=68% |
| 53 | LARSSON\_STATE\_1 |  | 28 | -0.40 | -1.80 | 0.029 | 0.020 | 0.633 | 139 | tags=36%, list=14%, signal=40% |
| 54 | NB\_KI\_CLUSTER2 |  | 42 | -0.36 | -1.79 | 0.013 | 0.020 | 0.645 | 380 | tags=69%, list=38%, signal=107% |
| 55 | IVY\_CTMVP |  | 17 | -0.46 | -1.77 | 0.017 | 0.023 | 0.701 | 209 | tags=59%, list=21%, signal=73% |
| 56 | RICHARDS\_VERHAAK\_CANCERCELL\_2010\_MESENCHYMAL |  | 22 | -0.43 | -1.75 | 0.033 | 0.026 | 0.754 | 198 | tags=50%, list=20%, signal=61% |
| 57 | RICHARDS\_NOWAKOWSKI\_SCIENCE\_2017\_NIN1\_UPREG |  | 32 | -0.38 | -1.74 | 0.006 | 0.027 | 0.768 | 287 | tags=53%, list=29%, signal=72% |
| 58 | SUVA\_MES2\_PMID\_31327527 |  | 17 | -0.45 | -1.71 | 0.022 | 0.032 | 0.844 | 194 | tags=53%, list=20%, signal=65% |
| 59 | RICHARDS\_NEFTEL\_CELL\_2019\_MES2 |  | 17 | -0.45 | -1.69 | 0.033 | 0.037 | 0.894 | 194 | tags=53%, list=20%, signal=65% |
| 60 | RICHARDS\_CAHOY\_JNEUROSCI\_2014\_MOG\_POS |  | 72 | -0.29 | -1.67 | 0.020 | 0.039 | 0.910 | 191 | tags=32%, list=19%, signal=37% |
| 61 | RICHARDS\_NOWAKOWSKI\_SCIENCE\_2017\_EN\_V1\_1\_UPREG |  | 131 | -0.25 | -1.67 | 0.010 | 0.039 | 0.913 | 430 | tags=61%, list=44%, signal=94% |
| 62 | FELDMAN\_2020\_LATEG1 |  | 49 | -0.32 | -1.67 | 0.023 | 0.040 | 0.917 | 124 | tags=29%, list=13%, signal=31% |
| 63 | RICHARDS\_CAHOY\_JNEUROSCI\_2012\_OPC |  | 338 | -0.22 | -1.66 | 0.000 | 0.040 | 0.922 | 161 | tags=22%, list=16%, signal=17% |
| 64 | RICHARDS\_NOWAKOWSKI\_SCIENCE\_2017\_NIN3\_UPREG |  | 25 | -0.40 | -1.66 | 0.032 | 0.039 | 0.922 | 225 | tags=52%, list=23%, signal=66% |
| 65 | RICHARDS\_CAHOY\_JNEUROSCI\_2009\_NEURON |  | 106 | -0.26 | -1.62 | 0.021 | 0.051 | 0.965 | 347 | tags=53%, list=35%, signal=73% |
| 66 | RICHARDS\_NOWAKOWSKI\_SCIENCE\_2017\_IN\_CTX\_MGE2\_UPREG |  | 32 | -0.35 | -1.61 | 0.045 | 0.052 | 0.967 | 310 | tags=59%, list=31%, signal=84% |
| 67 | RICHARDS\_NOWAKOWSKI\_SCIENCE\_2017\_IN\_STR\_UPREG |  | 25 | -0.37 | -1.58 | 0.058 | 0.061 | 0.979 | 380 | tags=72%, list=38%, signal=114% |
| 68 | RICHARDS\_NOWAKOWSKI\_SCIENCE\_2017\_EN\_PFC1\_UPREG |  | 122 | -0.24 | -1.56 | 0.022 | 0.067 | 0.985 | 368 | tags=53%, list=37%, signal=74% |
| 69 | LARSSON\_STATE\_5 |  | 22 | -0.38 | -1.55 | 0.057 | 0.070 | 0.991 | 212 | tags=45%, list=21%, signal=57% |
| 70 | RICHARDS\_CAHOY\_JNEUROSCI\_2016\_CULTURED\_ASTROGLIA |  | 224 | -0.22 | -1.55 | 0.022 | 0.070 | 0.993 | 180 | tags=25%, list=18%, signal=24% |
| 71 | ALMSTEDT\_11Q\_MSIGDB |  | 23 | -0.36 | -1.51 | 0.075 | 0.083 | 0.996 | 115 | tags=30%, list=12%, signal=34% |
| 72 | RICHARDS\_NOWAKOWSKI\_SCIENCE\_2017\_IN\_CTX\_MGE1\_UPREG |  | 45 | -0.29 | -1.50 | 0.076 | 0.087 | 0.997 | 267 | tags=44%, list=27%, signal=58% |
| 73 | FELDMAN\_2020\_LOWRNA |  | 209 | -0.21 | -1.50 | 0.035 | 0.086 | 0.997 | 154 | tags=22%, list=16%, signal=20% |
| 74 | DOROSZKO\_U3054MG\_CLUSTER\_5 |  | 22 | -0.35 | -1.47 | 0.090 | 0.103 | 0.999 | 367 | tags=73%, list=37%, signal=113% |
| 75 | RICHARDS\_NOWAKOWSKI\_SCIENCE\_2017\_IPC\_NEN1\_UPREG |  | 40 | -0.29 | -1.46 | 0.100 | 0.105 | 0.999 | 294 | tags=52%, list=30%, signal=72% |
| 76 | FELDMAN\_2020\_M-EARLYG1 |  | 52 | -0.27 | -1.46 | 0.063 | 0.104 | 0.999 | 111 | tags=23%, list=11%, signal=25% |
| 77 | FELDMAN\_2020\_NEURAL G0 |  | 139 | -0.22 | -1.44 | 0.062 | 0.110 | 1.000 | 92 | tags=16%, list=9%, signal=15% |
| 78 | GANDAL\_ALCOHOLADDICTION\_DN\_PMID29439242 |  | 15 | -0.40 | -1.43 | 0.108 | 0.114 | 1.000 | 230 | tags=53%, list=23%, signal=68% |
| 79 | RICHARDS\_NOWAKOWSKI\_SCIENCE\_2017\_EN\_V1\_3\_UPREG |  | 119 | -0.22 | -1.43 | 0.067 | 0.115 | 1.000 | 360 | tags=50%, list=36%, signal=70% |
| 80 | RICHARDS\_NOWAKOWSKI\_SCIENCE\_2017\_IPC\_NEN1\_UPREG\_1 |  | 20 | -0.35 | -1.40 | 0.136 | 0.133 | 1.000 | 249 | tags=50%, list=25%, signal=65% |
| 81 | ALMSTEDT\_17Q\_MSIGDB |  | 26 | -0.33 | -1.39 | 0.128 | 0.136 | 1.000 | 4 | tags=8%, list=0%, signal=8% |
| 82 | RICHARDS\_NOWAKOWSKI\_SCIENCE\_2017\_NEN\_EARLY2\_UPREG |  | 63 | -0.25 | -1.39 | 0.092 | 0.135 | 1.000 | 235 | tags=38%, list=24%, signal=47% |
| 83 | RICHARDS\_NOWAKOWSKI\_SCIENCE\_2017\_EN\_V1\_2\_UPREG |  | 110 | -0.21 | -1.37 | 0.096 | 0.149 | 1.000 | 340 | tags=46%, list=34%, signal=63% |
| 84 | RICHARDS\_NOWAKOWSKI\_SCIENCE\_2017\_CHOROID\_UPREG |  | 85 | -0.22 | -1.34 | 0.118 | 0.163 | 1.000 | 220 | tags=33%, list=22%, signal=39% |
| 85 | NB\_KI\_CLUSTER4 |  | 23 | -0.32 | -1.33 | 0.165 | 0.174 | 1.000 | 275 | tags=48%, list=28%, signal=65% |
| 86 | DOROSZKO\_U3054MG\_CLUSTER\_10 |  | 37 | -0.27 | -1.32 | 0.150 | 0.179 | 1.000 | 225 | tags=38%, list=23%, signal=47% |
| 87 | KRONA\_ROSEN\_WHITE\_MATTER\_INVASION |  | 34 | -0.26 | -1.26 | 0.218 | 0.228 | 1.000 | 300 | tags=44%, list=30%, signal=61% |
| 88 | RICHARDS\_CAHOY\_JNEUROSCI\_2013\_OL\_MYEL |  | 112 | -0.20 | -1.26 | 0.161 | 0.226 | 1.000 | 325 | tags=44%, list=33%, signal=58% |
| 89 | GAROFANO\_IAVARONE\_NEU\_SUBTYPE |  | 27 | -0.28 | -1.25 | 0.219 | 0.235 | 1.000 | 132 | tags=30%, list=13%, signal=33% |
| 90 | RICHARDS\_NOWAKOWSKI\_SCIENCE\_2017\_NIN4\_UPREG |  | 29 | -0.27 | -1.22 | 0.242 | 0.261 | 1.000 | 219 | tags=38%, list=22%, signal=47% |
| 91 | DOROSZKO\_U3054MG\_CLUSTER\_9 |  | 54 | -0.22 | -1.19 | 0.230 | 0.289 | 1.000 | 184 | tags=26%, list=19%, signal=30% |
| 92 | KLING\_GBM\_PHENO\_PMID\_27667176 |  | 33 | -0.25 | -1.19 | 0.260 | 0.286 | 1.000 | 167 | tags=30%, list=17%, signal=35% |
| 93 | NB\_KI\_CLUSTER16 |  | 19 | -0.30 | -1.15 | 0.321 | 0.331 | 1.000 | 279 | tags=53%, list=28%, signal=72% |
| 94 | RICHARDS\_NOWAKOWSKI\_SCIENCE\_2017\_IPC\_NEN3\_UPREG |  | 50 | -0.21 | -1.12 | 0.311 | 0.363 | 1.000 | 256 | tags=40%, list=26%, signal=51% |
| 95 | RICHARDS\_NOWAKOWSKI\_SCIENCE\_2017\_NIN5\_UPREG |  | 45 | -0.21 | -1.10 | 0.337 | 0.388 | 1.000 | 346 | tags=51%, list=35%, signal=75% |
| 96 | TF\_CENSUS\_TAIPALE |  | 88 | -0.18 | -1.09 | 0.350 | 0.394 | 1.000 | 275 | tags=36%, list=28%, signal=46% |
| 97 | RICHARDS\_NOWAKOWSKI\_SCIENCE\_2017\_IN\_CTX\_CGE1\_UPREG |  | 41 | -0.22 | -1.07 | 0.368 | 0.410 | 1.000 | 220 | tags=32%, list=22%, signal=39% |
| 98 | RICHARDS\_NOWAKOWSKI\_SCIENCE\_2017\_NEN\_LATE\_UPREG |  | 42 | -0.21 | -1.05 | 0.417 | 0.436 | 1.000 | 360 | tags=52%, list=36%, signal=79% |
| 99 | NB\_KI\_CLUSTER6 |  | 29 | -0.22 | -0.99 | 0.471 | 0.512 | 1.000 | 239 | tags=38%, list=24%, signal=49% |
| 100 | RICHARDS\_NOWAKOWSKI\_SCIENCE\_2017\_GLYC\_UPREG |  | 82 | -0.16 | -0.97 | 0.500 | 0.541 | 1.000 | 301 | tags=35%, list=30%, signal=47% |
| 101 | DOROSZKO\_U3054MG\_CLUSTER\_3 |  | 34 | -0.20 | -0.93 | 0.560 | 0.595 | 1.000 | 221 | tags=35%, list=22%, signal=44% |
| 102 | RICHARDS\_INHOUSE\_BULKRNASEQ\_2019\_DEVELOPMENTALGSC |  | 177 | -0.13 | -0.93 | 0.596 | 0.595 | 1.000 | 109 | tags=13%, list=11%, signal=12% |
| 103 | RICHARDS\_NOWAKOWSKI\_SCIENCE\_2017\_MGE\_IPC3\_UPREG |  | 27 | -0.21 | -0.91 | 0.557 | 0.614 | 1.000 | 414 | tags=59%, list=42%, signal=99% |
| 104 | RICHARDS\_NOWAKOWSKI\_SCIENCE\_2017\_NIN2\_UPREG |  | 61 | -0.16 | -0.89 | 0.603 | 0.639 | 1.000 | 164 | tags=23%, list=17%, signal=26% |
| 105 | RICHARDS\_NOWAKOWSKI\_SCIENCE\_2017\_U4\_UPREG |  | 135 | -0.11 | -0.76 | 0.827 | 0.812 | 1.000 | 130 | tags=16%, list=13%, signal=16% |
| 106 | DOROSZKO\_U3054MG\_CLUSTER\_8 |  | 44 | -0.14 | -0.72 | 0.819 | 0.850 | 1.000 | 25 | tags=5%, list=3%, signal=4% |
| 107 | RICHARDS\_NOWAKOWSKI\_SCIENCE\_2017\_U3\_UPREG |  | 83 | -0.10 | -0.61 | 0.931 | 0.936 | 1.000 | 80 | tags=10%, list=8%, signal=10% |
| 108 | RICHARDS\_CAHOY\_JNEUROSCI\_2008\_OLIGODENDROCYTE |  | 145 | -0.09 | -0.58 | 0.969 | 0.944 | 1.000 | 273 | tags=30%, list=28%, signal=35% |
Table: Gene sets enriched in phenotype **na **[plain text format]****
